# Environmental Risk Factors for Multiple Sclerosis: A Review with a Focus on Molecular Mechanisms

**DOI:** 10.3390/ijms130911718

**Published:** 2012-09-18

**Authors:** Cullen O’Gorman, Robyn Lucas, Bruce Taylor

**Affiliations:** 1 School of Medicine, Griffith University, Gold Coast, Queensland 4222, Australia; 2 National Centre for Epidemiology and Population Health, the Australian National University, Canberra, Australian Capital Territory 0200, Australia; E-Mail: robyn.lucas@anu.edu.au; 3 Menzies Research Institute Tasmania, University of Tasmania, Hobart, Tasmania 7000, Australia; E-Mail: bruce.taylor@utas.edu.au

**Keywords:** multiple sclerosis, demyelination, epidemiology, latitude, vitamin D, Epstein-Barr virus, smoking, gene-environment interaction

## Abstract

Multiple sclerosis (MS) is a chronic disabling disease of the central nervous system commonly affecting young adults. Pathologically, there are patches of inflammation (plaques) with demyelination of axons and oligodendrocyte loss. There is a global latitude gradient in MS prevalence, and incidence of MS is increasing (particularly in females). These changes suggest a major role for environmental factors in causation of disease. We have reviewed the evidence and potential mechanisms of action for three exposures: vitamin D, Epstein Barr virus and cigarette smoking. Recent advances supporting gene-environment interactions are reviewed. Further research is needed to establish mechanisms of causality in humans and to explore preventative strategies.

## 1. Introduction

There is increasing evidence that a number of environmental factors are important in the development and course of multiple sclerosis (MS). MS is a chronic disabling disease of the central nervous system (CNS) that characteristically follows a waxing and waning course over many years before progressive disability supervenes. Pathologically, there are patches of inflammation within the CNS (plaques) with demyelination of axons and oligodendrocyte loss. Neuronal death (axon loss) is present early in the disease course, but becomes the predominant feature as the disease develops over time. It is hypothesized that loss of axons is the main mechanism underlying progressive disability [1].

Treatments have been available for MS since the late 1980s, based around immune modulation and immune suppression strategies. Whilst effective, these treatments do not stop the inflammatory process within the CNS seen on magnetic resonance imaging (MRI). Disease modifying therapies may be more effective if started earlier in the course of the disease and the diagnostic criteria have been recently revised to recognize the importance of early treatment [2]. Recent years have seen the introduction of more potent agents with greater potential to slow or cease the inflammatory process within the CNS. However, new agents have been associated with risk of adverse events including death. There remains no effective treatment for the progressive stage of the disease [3].

## 2. Epidemiology of MS

The prevalence of MS has been recorded as >200/100,000 in restricted populations, where artificially high ascertainment inflates estimates [4,5], but is at least 100/100,000 in Canada and higher in the Scandinavian countries. There is a global latitude gradient with lower prevalence seen nearer the equator [6]. In some isolated communities there is “resistance” to MS in otherwise high prevalence areas (e.g., the Sámi in Scandinavia) [7]. Generally MS is of lower prevalence in Asian countries and is more common within populations as socioeconomic status increases [8,9].

There has been a general increase in disease prevalence in the last few decades that cannot be attributed to advances in neuroimaging or changes in diagnostic criteria [10]. Early authorities considered the incidence to be equal in females and males [11] but there is a slight female predominance in most prevalence studies [6]. A trend for increasing female predominance over calendar time has been reported in several regions [12–17]. In a recent meta-analysis, sex ratio decreased with increasing latitude [16] and a similar finding was seen within a single study in a relatively homogeneous population [18]. This may reflect a differential effect of latitude-related factors on MS risk by gender [6,18], or changes in gender-specific smoking habits [19]. In a recent study of the US military veteran population, MS incidence was three-fold higher in women than in men, and incidence rates for Blacks were higher than for Whites (Relative risk (RR) = 1.27, 95% CI 1.16–1.39), over a 60-year time period [20].

Migrant studies further support the important influence of environmental factors in risk of MS. In a systematic review of such studies [21] two consistent patterns were apparent: migrants moving from a region of high MS risk to one of lower risk had a lower-than-expected MS prevalence, particularly when migration occurred before age 15 years; migrants moving from an area of lower risk to one of higher risk tended to retain the lower MS risk of their country of origin, with no clear age-at-migration effect. More recent studies have largely confirmed these patterns [22–28].

A large number of aetiological factors have been identified to play a role in MS including genetic susceptibility, smoking [29], exposure to the Epstein-Barr virus (EBV) [30] and low exposure to sunlight (presumed to be mediated through vitamin D insufficiency) [31,32]. These factors are reviewed below.

## 3. Genetic Risk

The evidence for genetic association is reviewed in this issue. For our purposes it is relevant to note an association between MS and the Major Histocompatibility Complex (MHC) (or human leucocyte antigen (HLA)) was first described in the 1970s [33]. Associations between HLA-DR15 haplotype (the DRB1*1501 allele and the alleles with which it is in linkage disequilibrium: DQA1*0102, and DQB1*0602) and MS have been described throughout European and non-European populations with MS [34]. The population of Sardinia is a notable exception, having an association with DR4 (DRB1*0405, DQA1*0301, DQB1*0302) [35,36] and not HLA-DR15 [37]. A number of other susceptibility loci outside the MHC have been identified through GWAS (genome-wide association study) and meta-analysis. Genetic modeling finds the largest effect at HLA-DR*1501 (Odds ratio (OR) = 3.2), with non-MHC susceptibility loci exerting modest effects (OR 1.1–1.3) and all loci acting independently [38–45]. Multiplex families seem to possess the same susceptibility genes as sporadic cases of MS, albeit in greater number [46,47]. It has been estimated that there are potentially 350 candidate susceptibility genes outside the MHC [48]. The most recent association studies find there are now over 50 genetic loci having confirmed association, with many other candidate loci identified at lower probabilities awaiting confirmation with larger scale studies [49,50], including genes involved in vitamin D metabolism [44,49]. However, the identified associations explain only a small fraction of the familial aggregation of MS [45].

Studies based upon familial aggregation and twin pairs have found increased risk to relatives with increased relatedness. Importantly, studies based on half-siblings [51,52], adoptees [53] and step-siblings [54] have found no effect of the family microenvironment on MS risk, suggesting that environmental factors act at a population level.

Risk to family members increases with increased latitude [55,56]. A recent systematic meta-analysis of family studies found risk to older relatives in particular (e.g., parents, aunts, uncles) seems most affected by latitude [57]. Overall genetic risk (as estimated by λ [58]) was stable against latitude. Modelling based upon this meta-analysis of family and twin data suggests a multiplicative model of genetic risk, with many (>1500) loci with small effect.

## 4. Environmental Risk Factors

### 4.1. Latitude

There is a distinct variation in MS prevalence with latitude. Latitudinal gradients have been identified throughout the world including Europe [59], North America [60,61], Australia [62], New Zealand [63] and Japan [64]. There was no significant gradient found in Canada [65] and Argentine Patagonia [66], although here the latitude range over populous regions was relatively small. Italy and the highest latitude regions of Scandinavia show an inverse gradient [67–69] although this neutralized when adjusted for the prevalence of HLA genotype variations between North and South [6]. There is a discrete focus of high prevalence in Sardinia and this has been suggested as due to genetic isolation and a founder effect [69,70]. As noted above, there is some evidence that twin concordance and recurrence risk within families may be influenced by latitude [55,57].

Accuracy of prevalence data may be influenced by changes in diagnostic criteria, the advent of MRI, longer life expectancy, and differing healthcare access. Analysis of disease incidence against latitude for the Northern Hemisphere, which may be less influenced by these factors, finds little association [15,16]. However, changes in disease incidence may also reflect changes in lifestyle and migration [71].

In Norway, the prevalence of MS is higher in inland farming areas than in fishing villages [72,73]. It has been proposed that at the highest latitudes, dietary sources of vitamin D such as oily fish may compensate for the relative lack of ultraviolet (UV) radiation exposure [74–76], although other factors, such as use of sunbeds, sunny holidays or taking vitamin D supplements could also contribute [77].

The first genetic and latitudinal influences on MS prevalence were detected by Davenport [78]. In a survey of men drafted to the US army he noted a prevalence for the US of 10/100,000 with higher prevalence in urban areas and among certain ethnic groups (Finns, Scandinavians). There was a latitudinal gradient with the highest prevalence seen in men from higher latitudes. It should be noted that racial distribution and latitude were not independent, with Finns and Scandinavians found most commonly at higher latitudes.

In retrospect, the findings of Acheson *et al*. [79] were particularly pertinent. They examined birthplace and place of residence of patients with MS discharged between 1954 and 1958. Only those hailing from counties with a population of >300,000 in 1920 were subjected to analysis (454 cases). They found significant correlations between disease prevalence and solar radiation (average annual sunshine and December solar radiation). Latitude was strongly correlated with MS prevalence, but did not have a significant independent effect.

Vitamin D was first proposed as an explanation for the latitude gradient by Goldberg [74]. The primary source of vitamin D in humans is UVB radiation (from sunlight) of the skin [80]. The intensity of UVB wavelengths that reach the Earth’s surface varies according to latitude and season. Assuming equal sun exposure to an equivalent amount of skin, lower intensity of UVB radiation in winter may be insufficient to support vitamin D synthesis in some locations. Vitamin D deficiency has been demonstrated in the United States [81] and Australia [82] for at least part of the year.

Despite considerable evidence for a role of vitamin D in MS pathogenesis (reviewed below), recent research has suggested that vitamin D status may not be the only mediator of a latitude effect related to exposure to UV radiation [83].

### 4.2. Vitamin D

“Vitamin D” is commonly used to refer to a group of fat soluble secosteroids. The active form, 1,25 dihydroxyvitamin D (1,25(OH)_2_D), has wide ranging effects in the human body, largely mediated by the action of 1,25(OH)_2_D on gene expression at the nuclear level.

The most recognized role is in calcium homeostasis, where 1,25(OH)_2_D and parathyroid hormone (PTH) act to maintain stable serum calcium levels through their action on bone, intestinal calcium absorption and renal calcium excretion [84]. However, more recently, evidence supports a role for 1,25(OH)_2_D in brain development and function, cardiovascular health, and musculoskeletal health. Furthermore, 1,25(OH)_2_D is thought to have anti-neoplastic properties, regulate insulin production and have extensive immunomodulatory effects [85].

Vitamin D has two main forms: ergocalciferol (vitamin D_2_) derived from plant matter and cholecalciferol (vitamin D_3_) derived from animal sources. Vitamin D_3_ is the primary vitamin D precursor in the human system. It is produced in the epidermis through cleavage of the β ring of the precursor, 7-dehydrocholesterol (pre-vitamin D_3_), by solar radiation in the UVB wavelengths [86,87] to form pre-vitamin D, which then spontaneously isomerises to vitamin D_3_. Both vitamin D_3_ (e.g., in oily fish) and vitamin D_2_ (e.g., in irradiated mushrooms) can be derived from dietary sources, but the latter is less efficiently absorbed than vitamin D_3_ and is less biologically active [88]. Once in the circulation vitamin D (both D_2_ and D_3_) is transported to the liver, where it is hydroxylated to the major circulating form, 25-hydroyvitamin D_3_ (25(OH)D). From the liver, 25(OH)D is transported tightly bound to the vitamin D binding protein (VDBP) to the kidneys and other target organs, where it is converted to the biologically active 1,25(OH)_2_D [89]. The active form has a short serum half-life due to tight regulation by the endocrine system to maintain calcium homeostasis—consequently serum 25(OH)D is used as the marker for vitamin D status with a 30 day serum half-life [89].

In the endocrine system 1,25(OH)_2_D is produced in the kidneys by the action of the 1α hydroxylase enzyme encoded by the *CYP27B1* gene on chromosome 12. In addition to this, there is local conversion of 25(OH)D to 1,25(OH)_2_D in a variety of cell and tissue types where the 1α hydroxylase enzyme is present in the mitochondria [90]. In contrast to the endocrine production of 1,25(OH)_2_D, which is tightly regulated by parathyroid hormone and calcium levels, local production is more dependent on the serum level of 25(OH)D and the local cytokine milieu [91]. Of particular relevance to MS, is that 1α hydroxylase is present in the mitochondria of many cell types of the immune system [78], as well as neuronal and glial cells of the CNS [90,92].

#### 4.2.1. Cellular and Immune Functions

Vitamin D can act upon target cells via two mechanisms: as 25(OH)D bound to the VDBP which can actively or passively cross the cell membrane and be converted to 1,25(OH)_2_D in the mitochondria of capable cells and then bind to the vitamin D receptor (VDR) to exert its genomic effects [93]; or as free 1,25(OH)_2_D—produced in the kidneys (endocrine), in other local cells (paracrine), or from the cell itself (autocrine)—which can bind to membrane based VDRs or cross the cell membrane and exert either rapid and intermediate, non-genomic effects or bind to cytoplasmic or nuclear VDRs to exert genomic effects.

The cell membrane VDR is responsible for the more rapid effects of 1,25(OH)_2_D [85], that begin within seconds of binding of 1,25(OH)_2_D to VDR [93,94]. Here the actions are principally at the level of the plasma membrane and include the modulation of ion or ligand-gated ion channels and the production of cyclic AMP or inositol-1,4,5-triphosphate at the cell membrane, affecting ion channels and membrane-bound signal-transduction. 1,25(OH)_2_D also has various intermediate effects [93,94], occurring hours after exposure, including activation of protein kinases. Some of the rapid effects of 1,25(OH)_2_D occur independently of the VDR thus providing the mechanism by which cells that do not manifest the VDR may be affected by endocrine or paracrine 1,25(OH)_2_D [95]. These rapid and intermediate effects, particularly the phosphorylative effects of activated protein kinases, act to modulate the genomic effects of 1,25(OH)_2_D [93,96].

1,25(OH)_2_D has been demonstrated *in-vitro* to modulate the immune response [97], depressing or inhibiting the production of pro-inflammatory T_h_1 cytokines while stimulating T-helper type 2 cell (T_h_2) and regulatory T cell (T_reg_) activity [98]. in the CNS, 1,25(OH)_2_D has been found to block the production of pro-inflammatory cytokines and nitric oxide by microglia [99]. Additionally, 1,25(OH)_2_D is necessary for neurotransmitter and neuronal function [100]. Recently, 1,25(OH)_2_D has been shown to moderate demyelination and potentiate remyelination, halt oligodendrocyte apoptosis, and stimulate the differentiation of oligodendrocyte precursors into mature cells [101].

#### 4.2.2. Genomic Effects of Vitamin D

Vitamin D exerts its genomic effects via the conversion of 25(OH)D to 1,25(OH)_2_D in the mitochondria, and interaction with VDR, in a highly ordered process that involves a chain of chaperones and co-chaperones [94]. The vitamin D–VDR complex undergoes heterodimerization with any of three retinoid X receptor (RXR) isoforms and binds to specific vitamin D responsive elements (VDREs) in the promoter region of target genes, up-regulating or repressing transcription [94,102]. VDREs are specific DNA sequences that recognize with high affinity the 1,25(OH)_2_D-VDR-RXR complex and have a highly conserved morphology [102]. Many other chaperones and co-activators or co-repressors may be involved and these complexes include an acetyltransferase component that contains members of the p160 family including SRC-1 (steroid receptor co-activators-1), TIF2 (transcriptional intermediary factor 2, and RAC3 (receptor activated co-activators) and p300/CBP as well as D receptor interacting protein (DRIP205) [103–105]. The local and cell and gene specific actions of these co-activators, co repressors and chaperones combine to determine whether the response to the complex binding to the VDRE enables opening of the chromatin structure to allow gene transcription or closes the chromatin to decrease gene expression.

Some of these genomic effects act to regulate vitamin D metabolism [106,107] but also affect a range of genes related to cellular proliferation/differentiation [108,109] and the immune system, including cytokines and cytokine receptors [109,110]. Ramagopalan and colleagues [111] recently demonstrated that the strongest MS genetic susceptibility region, the HLA-DRB1*1501 allele of the human leukocyte antigen-DR (*HLA-DR*) gene, is up-regulated by 1,25(OH)_2_D via a highly conserved VDRE.

#### 4.2.3. Evidence that Vitamin D Has a Role in MS

##### 4.2.3.1. Epidemiological Evidence

This molecular basis for a possible beneficial role of vitamin D in multiple sclerosis is supported by observational epidemiological studies. Two prospective studies have shown that low vitamin D intake [112] or serum vitamin D status [113] are associated with increased risk of developing MS. In a prospective study of sera collected by the US Department of Defense, there were fewer cases of MS observed in individuals within the highest quintile of 25(OH)D levels when compared to those in the lowest quintile [113]. Serum circulating 25-hydroxyvitamin D (25(OH)D) levels are lower in MS patients [114–117], but this is easily attributable to reduced activity levels consequent to disease [118]. However, MS patients have lower levels of vitamin D at time of relapse [116] and are at higher risk of relapse when serum levels are low [119–122].

##### 4.2.3.2. Genetic Evidence

Not unexpectedly, polymorphisms in the genes that encode the key proteins in the actions of vitamin D, including VDR (*VDR*) and *CYP27B1* that encodes 1α-hydroxylase, have shown evidence of association with the risk of developing MS. Polymorphisms in *CYP27B1* have been associated with a significantly increased risk of developing MS in some [49,123,124] but not all [125,126] studies. The evidence for an association between the development of MS and polymorphisms in the *VDR* is mixed, with some finding an association [127–129] but others finding no association [125,126,130–132]. In addition, interactions between *VDR* polymorphisms and *HLA-DR15* [133], exposure to sunlight [130] or dietary vitamin D intake [126] have been observed, but these have not yet been confirmed. However 1,25(OH)_2_D has pleotropic effects in the human genome as demonstrated by the findings of Ramagopolan *et al*. [134] who in lymphoblastoid cell lines (LCLs) determined VDR binding throughout the human genome using chromatin immunoprecipitation followed by massively parallel DNA sequencing (ChIP-seq). After stimulation with 1,25(OH)_2_D_3_, they identified 2776 genomic positions occupied by the VDR and 229 genes with significant changes in expression in response to 1,25(OH)_2_D. VDR binding sites were significantly enriched near autoimmune and cancer associated genes identified from GWAS. In particular VDREs and significant changes in gene expression were noted for around 80% of genes associated with MS at that time. Recently Disanto *et al*. [135] in LCLs found that VDR-binding regions overlapped with active regulatory regions (active promoter (AP) and strong enhancer (SE)) in LCLs more than expected by chance (45.3-fold enrichment for SE (*p* < 2.0 × 10^−5^) and 63.41-fold enrichment for AP (*p* < 2.0 × 10^−5^)). Around 77% of VDR regions were covered by either AP or SE elements. VDR binding also occurred more commonly than expected within MS associated genetic regions (3.7-fold enrichment, *p* < 2.0 × 10^−5^). This indicates that vitamin D may enhance or repress important genes that regulate proteins important in immune responses associated with the development of MS. It also indicates that genetic variability in these genes may influence the way that vitamin D may produce its effects on MS.

Providing further evidence that low 1,25(OH)_2_D levels may have an important influence on MS risk is the recent discovery in multiplex Canadian families of the first rare variants within the *CYP27B1* gene that are associated with an increased risk of MS onset (Peto OR = 4.7 (95% confidence interval CI, 2.3–9.4; *p* = 5 × 10^−7^)) [136]. The genetic mutations described in the homozygous state are associated with the development of vitamin D dependent rickets type 1 (VDDR1). Interestingly in all three cases of VDDR1 found in Norway there was a 100% concordance with MS [137] a highly statistically unlikely scenario by chance given the rate of VDDR1 is 1:500,000 and the rate of MS is 1:1000. In total Ramagopolan *et al*. identified 3 functional rare variants in *CYP27B1* that were associated with MS. In all cases the variants are associated with low 1,25(OH)_2_D levels and provide a strong link between low 1,25(OH)_2_D and risk of MS.

### 4.3. Epstein-Barr Virus

Many infections have been proposed to play a role in MS pathogenesis, with the most consistent findings in relation to past infection with Epstein Barr virus (EBV). Infection with this herpes virus is most often asymptomatic in childhood but in adolescence and adulthood it is commonly symptomatic, causing infectious mononucleosis (IM), which can be severe. In under-developed regions, most children become seropositive within the first ten years of life (>90% by 6–8 years of age in Bangkok [138]), while in developing countries delayed infection is common, with only 50% seropositive by age 5–9 years, but over 80% seropositive by adulthood [139]. It is this late infection with EBV, and a past history of IM, that seems particularly associated with increased MS risk [140,141].

As with MS, IM is uncommon in low latitude regions—but here the association may be more strongly related to socioeconomic rather than latitudinal factors [142]. For example, within a high latitude region, Greenland, IM was uncommon in the Indigenous population compared to the non-Indigenous population [143], highlighting the importance of social conditions. MS is uncommon in Asian populations and in early studies, IM was also uncommon [144], with high prevalence of seropositivity in childhood (>80% at 5–7 years in Tokyo prior to 1990), *i.e.*, early rather than delayed primary infection [139]. However in more recent years, this is changing, with a later age of acquiring EBV seropositivity, *i.e.*, 59% of 5–7 year olds in Tokyo were seropositive during 1995–1999. At the same time, MS is becoming more common in Japan [145].

At an individual-level, both case-control and cohort studies consistently report an increased risk of MS in association with past history of IM, or higher levels of EBV-specific antibodies (reviewed in [142]). For example, in a recent meta-analysis of 18 case-control and cohort studies a history of IM was associated with a two-fold increased risk of MS (OR = 2.17, 95% CI 1.97–2.39) [30]. Similarly, other meta-analyses reported a summary OR of 13.5 (95% CI 6.3–31.4) for MS risk in relation to seropositivity (compared to being seronegative, 8 studies) [146] and of decreased MS risk in association with EBV seronegativity (*vs*. being seropositive, 22 adult and 3 paediatric studies). For the latter, the assay used to measure EBV antibody titres was important, but when two independent methods were used (ELISA and immunofluorescence, 3 studies), the OR was 0.00 (95% CI 0–0.43), suggesting that EBV antibodies are present in 100% of MS patients [147]. In one recent study, presence of antibodies to the EBNA-1 fragment 385–420 was more strongly associated with MS risk than that of total EBNA-1 IgG (OR = 3.60, 95% CI 2.75–4.72, *vs*. 1.74, 95% CI 1.38–2.18) [148].

Most importantly, in large cohort studies serial blood samples prior to MS onset have shown that EBV seropositivity and elevation of EBV-specific antibody titres precedes disease onset, by at least several years [149–152]. In one study, of participants who were EBV seronegative on the first blood sample, 100% of cases seroconverted prior to MS onset, compared to only 35.7% of controls (consistent with seroconversion rates in healthy populations of a similar age) over the same period (*p* < 0.0001) [153].

In general, the increased MS risk is specifically associated with higher IgG antibody titres to Epstein-Barr nuclear antigens (EBNA) (including the nuclear antigen complex, and subtypes-1 and -2) with little association with antibodies to other EBV antigens or to other viral infections including to measles, HSV, VZV [152] or CMV [149,150]. These findings support a specific immune reaction to EBV, particularly EBNA, rather than a non-specific immune dysregulation associated with the disease itself.

#### 4.3.1. Interactions with Other Environmental and Genetic Factors

A number of environmental factors are thought to influence EBV infection or MS risk. One study reported an interaction between smoking and anti-EBNA IgG titres: the increased MS risk associated with higher anti-EBNA titers was stronger among ever smokers than never smokers (*p* for interaction =0.001); but the increased MS risk associated with smoking was seen ***only*** among those who had high anti-EBNA titers [154]. Nevertheless, no interaction, [155] and a negative interaction whereby the increased MS risk in association with a past history of IM was significantly higher among *non-smokers* (pooled OR = 2.4, 95% CI 1.8–3.2) compared to smokers (pooled OR = 1.6, 95% CI 1.3–2.1), *p* interaction = 0.04), has also been reported [156].

A growing body of evidence implicates vitamin D deficiency as a risk factor for MS (see previous section). Vitamin D has immunomodulatory effects on both the innate and adaptive immune systems that could have implications for the risk, severity or persistence of EBV infection. One study has reported an inverse correlation between serum 25(OH)D levels and EBV DNA load in people without MS [157] but there is not yet evidence of a significant interaction between measures of EBV infection and vitamin D deficiency that is associated with increased MS risk.

There is some evidence to suggest that the pattern of viral infections or co-infection with different viruses or different EBV types may be important. EBV infection without the protective benefit of earlier infection with another virus, e.g., herpes simplex virus may increase MS risk, [158,159] while subsequent infection, e.g., with HHV-6 variant A may result in reactivation of latent EBV infection thereby increasing MS risk [150]. EBV infection can transactivate human endogenous retroviruses (HERVs) in *in vitro* models [160] and some specific HERVs are implicated in MS risk [161]. Santon *et al*. reported that EBV types 1 and 2 were simultaneously detected in 63 out of 70 MS patients positive for EBV (90%), compared to 46 out of 123 positive controls (37%) [162].

Although interacting biological pathways involving the main susceptibility gene, HLA DRB1*15 as part of the MHC class II that presents pathogens to CD4+ cells, and EBV infection might be expected to increase MS risk, this has not been confirmed in empirical studies. Higher anti-EBNA-1 levels and HLA-DRB1*15 positivity appear to be independent risk factors for MS, [163,164] although their co-occurrence results in a marked (additive) increase in MS risk (e.g., 10-fold) compared to not having either [148,163]. Individuals positive for EBNA-1 fragment 385–420, positive for HLA-DRB1*15 and negative for HLA-A*02 had a 16-fold increased risk of MS (OR = 16.03, 95% CI 9.42–27.20) [148].

#### 4.3.2. Causal Pathways

There is little evidence to support that recent infection or reactivation with EBV causes MS: EBV DNA load is not consistently increased in peripheral blood in people with recently diagnosed MS [165–167] or a first clinical diagnosis of CNS demyelination [157,168] and evidence from seroconversion suggests new infection 5 or more years prior to MS diagnosis.

The hygiene hypothesis was first proposed in 1989 to explain the inverse association between larger family size and risk of hayfever [169] but is now more generally considered a possible explanation for a number of diseases of immune dysfunction including allergy and autoimmune diseases. Pre-dating Strachan’s work in allergy, Poskanzer proposed that MS might be caused by a poorly targeted and over-reactive immune response to a common childhood infectious agent that was encountered for the first time in later life [170]—a possible consequence of a more “hygienic” early life environment [171]. The core concept here is that exposure to microbial antigens in early life assists the correct development of a balanced immune system [172], in particular balance between the various types of T helper (T_h_) cells and T regulatory cells involved in the adaptive immune response [172]. MS is considered to involve over-reactivity of T_h_1 immune function [173] and upregulation of T_h_17 cells [172] with reduction in T regulatory cell activity [174].

Repeated exposure to a microbe results in a rapid and specifically targeted immune response—the basis of immunological memory. Recent evidence supports that in MS there is broadened specificity in the T cell response to EBNA-1 compared to controls [165,175,176] and EBNA-1 specific T cells were cross-reactive with myelin antigens [176]. A number of studies have shown that MS patients produce antibodies primarily to specific peptide regions of the EBNA-1 protein that are not significantly bound by healthy EBV seropositive controls but may be cross-reactive with myelin basic protein [148,177–179].

There is, nevertheless, some evidence that EBV infection is directly involved in the etiology of MS. Some studies have reported detection of EBV infection in a large fraction of the B cells infiltrating the MS brain [180,181], but this has not be confirmed in other studies [182,183]. A review of the conflicting findings concluded that “independent confirmation of the presence of a multiple sclerosis associated EBV infection in the CNS had so far not been achieved” [184]. Control of EBV infection involves the presentation of viral peptides by MHC class I molecules to CD8^+^ T cells, and by MHC class II molecules to CD4^+^ T cells. Both decreased CD8^+^ T-cell reactivity to EBV-infected B cells in patients with MS [185] and a significant *increase* in the frequency of CD8^+^ T cells specific for EBV proteins expressed in infected B cells [186], have been reported. The former could potentially allow accumulation of EBV-infected autoreactive B cells in the CNS [185], while expression of B-cell activating factor in MS lesions and B cell follicles could contribute to persistence of EBV in the CNS and contribute to MS development [181]. In one recent paper, EBV-specific CD8^+^ T cells were increased compared to healthy controls in early MS but not in more established disease [187]. Indeed the EBV-specific CD8^+^ T cell response was inversely proportional to the time from disease onset [187].

Exposure to infectious agents, and particularly EBV, results in the release from lymphoid cells of a small heat shock protein, αB-crystallin [188], which is followed by its HLA-DR restricted presentation to CD4^+^ T-cells and a proinflammatory T_h_1 response. The limited expression of αB-crystallin in human tissues, including the thymus is thought to underlie this failure of immune tolerance. αB-crystallin is a dominant myelin antigen in the human CNS [189] and accumulation of αB-crystallin in oligodendrocytes is observed in early MS lesions [190]. The pro-inflammatory response is thus thought to target oligodendrocytes and myelin, with resultant inflammatory demyelination [191].

Other hypotheses involve common pathways for EBV infection and lower sun exposure and/or vitamin D through alteration of levels or activity of IL-10. Sun exposure and vitamin D increase IL-10 levels, while EBV infection results in the production of a viral analogue of human IL-10 that may interfere with the normal actions of this cytokine that is produced by regulatory T and B cells and directly promotes neuronal and glial cell survival [192]. Pender has proposed an hypothesis for the more general development of autoimmunity, whereby in genetically susceptible individuals, EBV-infected autoreactive B cells seed a target organ, producing pathogenic autoantibodies and providing co-stimulatory survival signals to autoreactive T cells which would otherwise die by activation-induced apoptosis [193]. There is some supporting evidence for this hypothesis in MS, including the presence of EBV-infected B cells in the CNS [180] and a beneficial response to B cell depletion with rituximab [194].

There is considerable evidence that EBV infection plays a role in the development of MS. That evidence suggests that “late” infection in adolescence or adulthood carries particular risks, although EBV is also implicated in pediatric MS. EBV seropositivity occurs years before the clinical onset of MS suggesting that direct infection is not causative. Nevertheless, it is difficult to be confident in drawing such a conclusion in the absence of prospective studies examining disease onset and timing of EBV infection.

### 4.4. Cigarette Smoking

Cigarette smoking is an important risk factor for MS given it may be relatively easily modified and could provide insights into the pathogenesis of the disease. The habit of cigarette smoking became widespread amongst men in the developed world by the middle of the 20th Century. From the 1960s onward, the prevalence of smoking in women increased as prevalence in men decreased, resulting in a trend towards an increasing female:male ratio in smokers, whilst smoking prevalence in the population as a whole declined [16,195–197].

In a large North American cohort, >50% of patients with MS were smokers or ex-smokers [198]. MS patients who smoke are heavier smokers than the general population or those with other chronic diseases [199,200] and they do not stop smoking after diagnosis [200,201].

#### 4.4.1. Epidemiological Evidence for Association

There is strong evidence from case-control studies that smoking is associated with MS. A meta-analysis of 14 studies found increased susceptibility to MS in smokers, risk ratio (RR) 1.48 (95% CI 1.35–1.63) [29]. There was evidence of a dose response gradient in several studies, but little consistency in quantifying tobacco exposure [202–205]. One study has suggested that any increased risk of MS associated with smoking diminishes after 5 years of abstinence [206]. In many studies, socio-economic status was not analyzed and confounding cannot be excluded. Smoking status was frequently self-reported. MS patients of lower socioecomonic status had a higher frequency of adverse health behaviors, including smoking [198]. Although self-reported smoking status can be reliable [154], accuracy of this measure is reduced in lower socioeconomic groups, younger ages and in some ethnic groups [207].

The changing sex-ratio of MS seen in many countries over the last few decades suggests an environmental exposure [14]. Sundstrom *et al*. [208] objectively measured tobacco exposure using plasma levels of cotinine—a nicotine metabolite, and found increased risk for females occurred at cotinine levels consistent with passive exposure to cigarette smoke. Two case-control studies found that passive exposure increased the risk of adult- and child-onset MS respectively [209,210]. The influence of maternal smoking habits is unclear with two case control studies finding no association [211] and higher risk [212].

It is relevant to note that studies of users of Swedish snuff, a tobacco product that provides users with a comparable nicotine dose to smoking, do not find increased risk of MS in users [206,213].

#### 4.4.2. Interaction with Other Risk Factors

A case-control study carried out on multiplex families for MS, using healthy siblings as controls, found no increased risk of MS with smoking [214], suggesting there is no interaction between susceptibility alleles for MS and cigarette smoke exposure. However, Hedstrom and colleagues found possible interactions between susceptibility alleles and smoking habit in a population-based case-control study [215]. They established the smoking habit, *HLA-DRB1* and *HLA-A* genotype of 843 cases of MS and 1209 controls. The presence of HLA-A*02 is known to reduce susceptibility to MS, although it is unclear whether this effect requires homozygosity. Taking HLA-A*02 negativity and HLA-DRB1*15 positivity as ‘genetic risk’, they found smokers with genetic risk to have OR 13.5 (95% CI 8.1–22.6) for having MS compared to non-smokers without genetic risk. Smokers without genetic risk had OR 1.4 (95% CI 0.9–2.1) and non-smokers with genetic risk had OR 4.9 (95% CI 3.6–6.6) when each was compared to non-smokers without genetic risk. Smokers with genetic risk had double the risk (a factor of 2.8 *vs*. 1.4) of smokers without genetic risk, and current smokers were at increased risk compared to ever-smokers. This result requires replication in other populations, since there were very few individuals in the stratified analyses, particularly in the genetic risk and smoking comparison (87 cases and 22 controls). Interactions between EBV and smoking are discussed above.

#### 4.4.3. Smoking and Progression of MS

Smoking is associated with increased risk of secondary progressive disease [216–220] and increases the risk of early progression from clinical isolated syndrome (CIS) to clinically definite MS [221]. Neuroimaging of MS patients found more active inflammation and greater brain atrophy in smokers [218,222] features associated with greater disability on long-term follow-up [223].

#### 4.4.4. Causal Pathways

Cigarette smoke contains thousands of components including particulate matter (tar), nicotine, carbon monoxide and other vapor phase components [224,225]. There may be up to 100,000 unidentified components [226]. Some of the known effects of these substances include pro-inflammatory actions, direct tissue damage and increased apoptosis, immunosuppressant and anti-oestrogen effects. There has been considerable speculation as to the role of individual components in the pathogenesis of MS.

The MS plaque of demyelination contains an inflammatory infiltrate predominantly composed of macrophages containing myelin protein fragments [227]. It has been assumed that the disease process is driven by T or B cells autosensitized to myelin components, although an alternative explanation is that primary injury to oligodendrocytes attracts local immune mechanisms including macrophages to the site of injury. Axon loss may be secondary to transection of axons within an inflammatory lesion [228] or result from direct insult.

##### 4.4.4.1. Effects on the Immune System

Cigarette smoking increases the risk of autoimmune diseases including rheumatoid arthritis [229] and systemic lupus erythematosus[230], and is associated with autoimmune disease in patients with multiple sclerosis [231].

Cigarette smoke acts on cellular and humoral components of the immune system, having proinflammatory and inhibitory effects [232,233] that may be reversible if exposure is ceased [232,234]. Current and past smokers have higher levels of fibrinogen, CRP and other inflammatory markers, higher levels of pro-inflammatory cytokines (e.g., IL-6) and fibrinogen levels correlated with exposure [235–239]. A sustained pro-inflammatory state could contribute to persistent autoimmunity.

Smokers suffer from more frequent and persistent acute respiratory infections [240]. Cigarette smoke alters antigen-mediated T-cell signaling [241], reduces clonal expansion and activation of CD4 T cells and reduces activation of CD8 T cells in response to infection [242]. Exposure to cigarette smoke also reduces the production of pro-inflammatory cytokines by antigen-presenting cells [243,244], decreases numbers of peripheral B cells and NK cells, and reduces serum immunoglobulin levels [234,245–247], other possible mechanisms underlying susceptibility to viral respiratory infection.

Respiratory infection is an important trigger for relapse in MS. Sibley *et al*. [248] found risk of relapse was increased almost 3-fold in the period 2 weeks prior and 3 weeks post-respiratory viral infection. This has been a consistent finding, with more relapses associated with infection in winter [249–251]. Tremlett *et al*. [251] found that erythemal UV radiation was a significant determinant of timing of relapse (as discussed earlier). Relapses related to infection seem to result in more permanent disability than non-infection related relapses [250].

The role of infection in causation or maintenance of autoimmune demyelination in MS is controversial. *Chlamydia pneumoniae* and Epstein Barr virus among other viruses have been suggested as possible suspect organisms. *C. pneumoniae* antibodies have been found in higher titre in smokers [252,253]. Patients with MS were more likely than controls to have evidence of active (perhaps chronic) infection with *C. pneumoniae* [254]. Sriram *et al*. [255] found a higher proportion of MS cases had CSF positivity for *C. pneumoniae*, although their results have not been easily replicated [256,257], and the role of *C. pneumoniae* remains a subject of debate [258].

The role of EBV infection in causation or maintenance of MS is reviewed in the previous section.

One theory of autoimmunity suggests that accumulation of cellular debris, perhaps from direct tissue damage by neurotoxic components of cigarette smoke, could overwhelm apoptotic mechanisms, leading to local immune reaction and sensitization to self-antigens. Smokers had an increased expression of Fas (CD95), a cell surface molecule on B cells and CD4 T cells that makes those cells more susceptible to apoptosis [259]. Increased burden of apoptosed material may be relevant if clearance mechanisms are impaired in individuals with autoimmunity.

There are effective mechanisms to prevent production of self-reactive T cells during maturation of the immune system. However, self-derived proteins may undergo a variety of post-translational modifications that increase their likelihood of generating autoimmunity, either through rendering them more amenable to uptake by antigen presenting cells or to presentation by class II HLA molecules [260,261]. Class II HLA molecules (-DQ, -DP, -DR) are found only on immune competent cells and have a specific role in initiating a general immune response to a foreign antigen. This role differs to Class I HLA molecules (-A, -B, -C) that are concerned with antigen derived from inside cells [262].

It has been demonstrated in rheumatoid arthritis that carriers of the HLA-DRB1*04 epitope who smoke are more likely to develop anti-cyclic citrullinated peptide (CCP) antibodies [263–268]. The risk increases with increasing smoke exposure and homozygosity for the HLA-DRB1*04 allele [268]. It is hypothesised that post-translational modification of this peptide may generate or drive a sustained autoimmune response [269]. The exact mechanisms by which cigarette smoke components influence this process are not understood. In humanized mouse models, it has been shown that a bacterial peptide (common to several bacteria) can induce cross-reactivity with a T cell receptor for myelin basic protein and result in an MS-like disease [270], despite binding with low affinity. This suggests that self-derived modified antigen or foreign antigens are capable of generating autoimmunity.

##### 4.4.4.2. Non-Immunological Effects

Penetration of the blood-brain barrier by activated lymphocytes, and consequent release of inflammatory mediators is a possible initiating event in the development of MS [271,272]. Studies in rats have demonstrated that nicotine acts directly upon small parenchymal microvessels [273] and tight junction proteins within the blood-brain barrier [274], increasing influx of permeable solutes [275] and changing blood flow to deep brain structures [276].

Cigarette smoke contains high concentrations of free radicals that may directly damage cells or DNA, possibly causing mutation or gene activation facilitating autoimmunity [277]. Neurons and oligodendrocytes have higher oxygen utilization than astrocytes, microglia or capillary endothelial cells and are more vulnerable to oxidative stress [278]. Cigarette smoke contains three gases implicated in oxidative injury to neural tissue: hydrogen cyanide, nitric oxide (NO), and carbon monoxide (CO).

Hydrogen cyanide is rapidly absorbed from the lungs following inhalation [279]. Cyanide toxicity results from impaired oxidative phosphorylation. Hydrogen cyanide binds with high affinity to the ferric ion within the haem component of cytochrome oxidase *a*_3_, disrupting structural integrity and activity of the enzyme. This prevents oxygen utilization by body tissues for the production of adenosine triphosphate [280].

Numerous animal experiments have produced demyelinating lesions in the CNS following exposure to cyanide in large doses (e.g., [281,282] and after chronic low dose exposure [283,284]. Demyelination may be more easily induced through repeated doses or chronic exposure to cyanide, than after a single massive dose [285] although Levine and Stypulkowski [280] found the respiratory route of administration allowed a single exposure to be titrated sufficiently to produce demyelinating lesions. Cyanide related CNS injury preferentially affects the corpus callosum [282], a region also involved in MS [228]. Patients with MS have no measurable abnormality of cyanide metabolism [286]. Cyanide ingestion in humans (seen with consumption of cassava—*Manihot Esculenta*) is associated with central and peripheral nervous system demyelinating disease [287–290].

NO has diverse biological roles including regulation of blood vessel tone, platelet aggregation, smooth muscle regulation, synaptic function, and innate immune system cytotoxicity [291]. Cigarette smoke contains many free radical derivatives of NO at concentrations up to 500 ppm [277,292]. Cigarette smoking is a major exogenous source of NO [293], and nicotine induces the production of NO in the CNS [294–296]. NO can inhibit mitochondrial chain components [297] and direct exposure can cause axonal degeneration or block nerve conduction, especially in physiologically active axons [298] or demyelinated axons [299]. Exposure to NO causes oligodendrocyte necrosis whilst astrocytes and microglia are unharmed [300–302].

CSF levels of NO or NO metabolites are increased during acute relapse [303–305]. And persistently elevated levels of NO metabolites in the CSF are associated with clinical progression of MS [306].

CO is a colorless, odorless gas produced by combustion in the absence of adequate oxygen. CO has a higher affinity than oxygen for binging to haemoglobin (230–260 fold) and is carried in the blood stream as carboxyhaemoglobin (COHb). Normal blood COHb levels of <1% are increased in smokers to >10% in some cases [307,308]. CO toxicity results from a combination of reduced oxygen delivery (through reduced available haemoglobin and shift of the oxyhaemglobin curve to the left); and direct competition with oxygen binding to cytochrome *a*_3_ [309,310]. Animal and human exposure to CO results in extensive cerebral demyelination [311–314].

There is substantial epidemiological evidence linking cigarette smoking with MS disease susceptibility and progression. Possible mechanisms for these effects include immunomodulation and direct toxicity to neurons and oligodendrocytes, but direct evidence linking smoking and MS is limited.

## 5. Summary

Geographic patterns of occurrence, changing sex ratios and increasing incidence over a relatively short time frame point to a major role for environmental factors in the causation of MS. In the face of imperfect and non-curative treatments, understanding the role and mechanisms of action of environmental exposures is highly important as these are potentially preventable. Here we have reviewed the evidence and possible routes of action of three of the best researched putative environmental risk factors for MS onset and progression. Most of the evidence derives from ecological and observational studies with support from animal experimental models and cellular studies. Clinical trials are now underway to establish whether vitamin D deficiency is truly a causal risk factor for MS. Such trials will not be possible for further examining Epstein Barr virus infection or smoking as risk factors. For the latter, the obvious course is to support ongoing health promotion efforts aimed at discouraging taking up smoking and encouraging smoking cessation. For Epstein Barr virus infection, further research is required to elucidate whether this is a marker, e.g., of co-infection or reactivation of another virus, and whether there is any place for preventive programs such as vaccination. The balance of risks and benefits of any such action will need to be fully understood prior to implementation.
